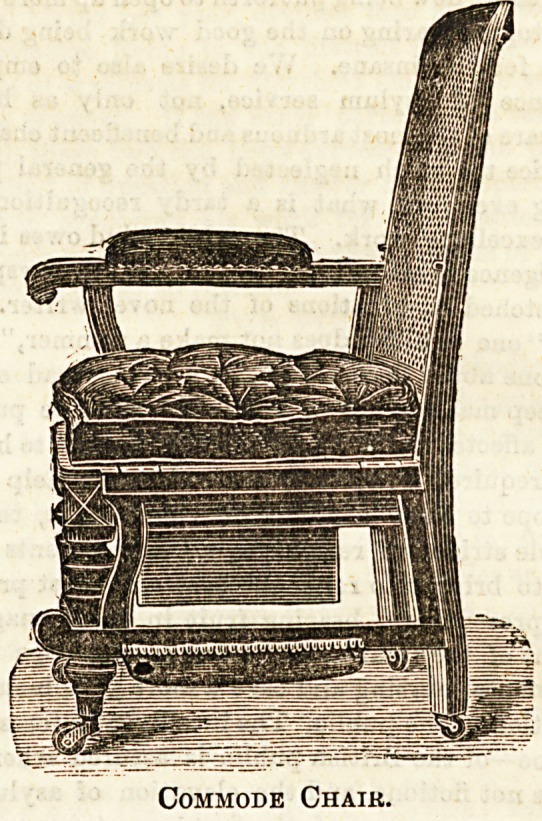# Carrying, Commode, and Bath Chairs

**Published:** 1892-05-14

**Authors:** 


### FURNITURE AND FITTINGS.

### CARRYING, COMMODE, AND BATH CHAIRS.

Within the last few years or so enormous strides have been made in the design of invalid chairs of all sorts. The directions in which improvements have manifested themselves lie, chiefly towards lightness combined with strength, portability and elegance. Messrs. Leveson and Sons, of New Oxford Street, have lately brought out some capital carrying chairs, which show many remarkable improvements on any of the old patterns which preceded them. They are designed chiefly for use in travelling, inasmuch as they can readily and easily

A, Carrying Chair as in use. B, Folded up.

Portable Carrying Chair.

be folded into a comparatively small compass, and then can be placed under the seat in a railway carriage, or on the top of a cab with the rest of the luggage. The legs are made to screw on and off, and the back and arms fold down quite flat, making a comparatively convenient package for an attendant to carry. One of the special features of these chairs is their extreme lightness, the framework being composed of birch, mahogany, or oak, the seat and back being fitted with fine cane work. If preferred, the whole chair can be produced in cane, but in this case the portability is somewhat sacrificed for the sake of lightness, as the legs are fixed and cannot be unscrewed; and it has the further disadvantage that the carrying poles pass right under the seat of the chair, and are consequently on the same level back and front, a serious inconvenience if the patient has to be carried about to any extent. This disadvantage cannot be urged against the chairs which

Commode Chair.

have wooden frames, as the carrying handles at the front of the chair, which are used by the rear bearer in going upstairs, and by the front bearer in going downstairs, are sufficiently below the level of the hind handles to make the process of carrying at once easy and comfortable.

These chairs, and the same may be said of most of those offered by Messrs. Leveson, are in themselves useful pieces of furniture, and may be used for ordinary sitting purposes, and they are by no means suggestive of the invalid room, but are rather ornamental than otherwise, and for the invalid they are sufficiently comfortable to be used as permanent seats, whether requiring to be carried about or not.

Some very convenient forms of commode chairs are also on view at Messrs. Leveson's, they are in themselves handsome pieces of furniture, and not in the slightest degree suggestive of the purpose for which they are intended, thus supplying a long-felt want in invalid rooms where visitors are received. The chairs are made of handsome carved walnut or mahogany, with double close cane seat and back, the arms are hinged, and can be let down. In many cases where the invalid has to be assisted by an attendant this is found to be a great convenience. The pan slips into a drawer which slides out at the back, and can consequently be placed in position without disturbing the patient. After the removal of the pan the draw slips back, and is held in position by a spring lock, and the chair has then the appearance of a well-made library chair. The cushion is made of tapestry, and the arm upholstered to match, and the legs are fitted with strong castors, so that the chair can readily be wheeled about the room. One of the chief advantages of these chairs is the fact that there is no possibility of any part of them becoming soiled during use. "Simplex munditiis," they are free from all complicated fittings and mechanism. It might, perhaps, be more convenient for the patient if the cushion could be removed from its position without disturbing the occupier; for instance, a central portion of the cushion might be drawn from behind. This might, however, introduce possibilities of soiling the upholstery. In certain cardiac cases where all exertions tell on the patient it would doubtless be very useful. Possibly the chair might be made of lighter construction and fitted with arms for carrying, as in the case of the chair above described, and in that case the drawer and pan would have to be carried separately.

In bath chairs there are also many improvements to be noticed on the cumbrous machines in use but a few years ago. Where a man was required ten years ago to move one of these chairs, a child can now do so with no exertion to itself. Manufacturers of these articles have not been slow to avail themselves of the improvements introduced by cycle makers, both with regard to the structure of the wheels and the india-rubber tyre with which they are surrounded.

View of Sink and Cistern.

Section.

Plan.

Bicycle wheels are invariably used, and probably soon by the adoption of cushion and pneumatic tyres the invalid will be saved the possibility of vibration. The self-steering front wheel has been adapted by Messrs. Leveson to all classes of wheel chairs, so that they can be pulled from in front by the guiding handle, or pushed from behind after the removal of the handle. In the latter case steering can be accomplished by a mere turn of the wrist by the person who is pushing. Another improvement, which has been introduced for wicker bath-chairs, is the removal of the usual raised ledge which borders the platform for the feet; this was a constant course of annoyance and danger to the invalid, both in getting in and getting out of the chair. Paralytics were liable to catch their feet, and always found difficulty in stepping out. The platform is now placed near the ground, and can be easily reached by the most gouty or rheumatic of patients.

## Figures and Tables

**Figure f1:**
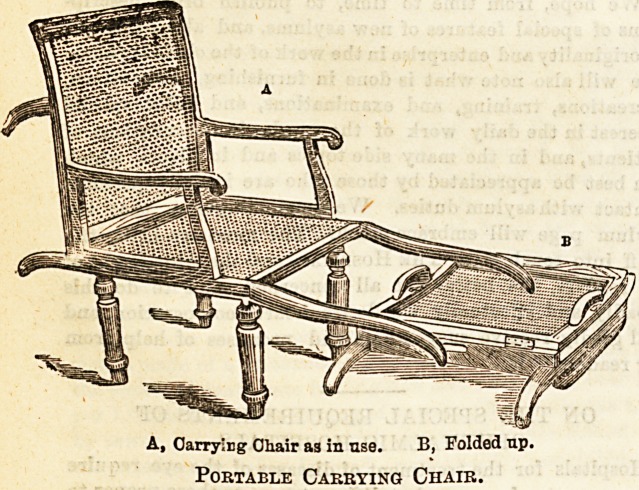


**Figure f2:**